# Management of Aortic Pseudoaneurysms: Evolving Concepts and Controversies

**DOI:** 10.1055/s-0039-1700999

**Published:** 2020-06-29

**Authors:** Sotiris C. Stamou, Brian D. Conway, Marcos A. Nores

**Affiliations:** 1 Department of Cardiothoracic Surgery, John Fitzgerald Kennedy Medical Center, Atlantis, Florida; 2 Department of Cardiothoracic Surgery, University of Iowa Hospitals and Clinics, Iowa City, Iowa

**Keywords:** aorta, pseudoaneurysms, outcomes, endovascular therapy, mortality, cardiac surgery, morbidity

## Abstract

**Background** Techniques to repair aortic pseudoaneurysms have been rapidly evolving. We present our results following open and endovascular repair of aortic pseudoaneurysms from 2009 to 2013.

**Methods** A total of nine patients underwent pseudoaneurysm repair from April 2009 to February 2013. Of them, five underwent open repair and four underwent endovascular repair. The median age was 58 years (range, 40–72 years) and two (22%) were females. Preoperative, operative, and postoperative data are presented along with operative modality.

**Results** Two patients died during the period of study. Patient 1 died from massive hemorrhage at the site of prior stenting. Patient 7 died from postoperative cardiac arrest and respiratory failure. A single patient required hemorrhage-related reexploration. None of the patients experienced stroke or acute renal failure following repair. Median hospital and intensive care unit length of stays were 7.1 (range, 1–20) and 2.0 (range, 1–5), respectively.

**Conclusions** Pseudoaneurysm repair can be effectively achieved through open or percutaneous repair but only after careful consideration of anatomical constraints, as well as patient comorbidities.

## Introduction

False aneurysms of the aorta, known as pseudoaneurysms, are hematomas formed outside the aortic wall, resulting from transmural disruption with the leak contained by surrounding mediastinal tissues. Previous cardiac surgery is the most frequent cause. 1 Typically pseudoaneurysms are a rare complication of aortic repair; however, in some studies, the incidence of pseudoaneurysm formation has been documented to manifest in as many as 7.7% of patients following aortic procedures. 2 Mechanisms implicated include infection, trauma, poor anastomotic technique, and intrinsic aortic wall disease. 1

Surgical treatments vary according to the features and locations of the pseudoaneurysm. Surgical challenges are frequent, especially in the presence of infection, prior cardiac surgery, and proximity of the pseudoaneurysm with the posterior sternal table making reentry particularly challenging. Less invasive options with coil embolization and pseudoaneurysm exclusion have also been reported. 3 4 5 Few studies have addressed the treatment of this complication. Herein, we describe our experience with endovascular and open surgical repair techniques for patients with pseudoaneurysms of the thoracic aorta.

## Materials and Methods

From April 2009 to February 2013, a total of nine patients underwent repair of pseudoaneurysms of the ascending and thoracic aorta ( Table 1 ). Preoperative, operative, and postoperative data were obtained by review of medical records. Mode of death was identified through review of clinical records and death certificates. Prior to this analysis, study approval from the Institutional Review Boards of each center was obtained. Consistent with the Health Insurance Portability and Accountability Act of 1996 (HIPAA), patient confidentiality was consistently maintained. Long-term survival data were obtained from the Social Security Death Index ( *http://search.ancestry.com/search/db.aspx?dbid=3693* ). Follow-up was 100% completed. Since social security death index has a 2-year blank out interval, we used obituary search where appropriate.

**Table 1:** Patient characteristics

Patient no.	Age (y)	Gender	Previous operation	Indication for repair	Operative interval (mo)	Repair date	Procedure	Outcome
1	65	Male	Bentall, March 2012	Bleed from distal suture line	2	May 2012	Redo the Bentall procedure with 29-mm valved conduit	Deceased in 2012 (bleeding from the native aorta at the previous stent site; cardiac arrest)
2	54	Male	Prosthetic aortic valve, January 2009	Endocarditis with pseudoaneurysm at aortotomy suture line	5	June 2009	Redo the Bentall procedure with 28-mm valved conduit	Alive
3	40	Male	Bentall, March 2007	Endocarditis with pseudoaneurysm at the distal suture line	63	June 2012	Redo the Bentall procedure with 25-mm valved conduit	Alive
4	60	Female	Ascending aorta replacement with aortic valve resuspension and CABG, April 2009; descending aorta replacement, September 2009; AVR, June 2011	Patient with Loeys–Dietz syndrome and pseudoaneurysm of both the proximal and distal suture lines	49	May 2013	Redo the Bentall procedure with bioprosthetic valve	Alive
5	68	Male	Ascending aorta replacement, June 2008	Pseudoaneurysm from distal suture line	32	April 2012	Resection of pseudoaneurysm under circulatory arrest	Alive
6	59	Male	CABG, November 2011	Ascending aortic pseudoaneurysm 2 cm above the RCA origin	20	December 2012	Six coil embolization wires, 10-mm Amplatzer atrial septal occluder device deployed under fluoroscopic guidance within the neck of the pseudoaneurysm	Alive
7	72	Male	Aorta to innominate and left subclavian bypass, 1992; pseudomonas graft infection requiring graft take-down, December 2007	Pseudoaneurysm from prior aortotomy site	55	June 2012	36 × 77 TX2 Cook thoracic endograft into the ascending aorta extending to the level of the left common carotid	Deceased (cardiac arrest; respiratory failure)
8	38	Female	Coarctation repair, 1978; MVA, 2007	Pseudoaneurysm at the previous repair site	412	April 2012	28 × 28 ×15 Tag (Gore) deployed in the descending aorta	Alive
9	68	Male	Coarctation repair, 1965	Pseudoaneurysm at the previous repair site	578	February 2013	28 × 10 Excluder (Gore) deployed in the descending aorta	Alive

Abbreviations: AVR, aortic valve replacement; CABG, coronary artery bypass grafting; MVA, motor vehicle accident; RCA, right coronary artery.

### Surgical Technique

Routine intraoperative transesophageal echocardiography was performed. Surgical technique varied based on the location of the pseudoaneurysm, history of previous cardiac surgery, proximity of the pseudoaneurysm with the posterior sternal table, presence of infection, and need for associated cardiac procedures. Median sternotomy was performed in five patients who underwent surgical repair of aortic root, ascending aorta, or aortic arch pseudoaneurysms. Arterial cannulation site was the femoral artery in three patients, axillary artery in one patient, and distal ascending aorta in one patient. Institution of cardiopulmonary bypass prior to sternal reentry was necessary in three patients to prevent accidental entry into the aneurysm because of proximity with the sternum. Cardiopulmonary bypass was instituted abruptly following pseudoaneurysm entry for one patient. Deep hypothermia and circulatory arrest was used in five patients, with antegrade cerebral perfusion in three patients. A small left anterior thoracotomy was performed in one patient to vent the left ventricle and prevent ventricular distention.

### Endovascular Repair

Endovascular repair was performed in four patients. Two patients had pseudoaneurysms of the descending thoracic aorta after repair of coarctation of the aorta and underwent repair with endovascular graft excluder of the pseudoaneurysms. One patient underwent aortic arch debranching and transaortic deployment of an endograft to exclude an arch pseudoaneurysm at a simultaneous setting. One patient with history of previous coronary bypass surgery developed a pseudoaneurysm at the aortic root vent site and underwent successful coil embolization and pseudoaneurysm exclusion.

## Results

### Preoperative Characteristics

A total of 22% of patients were females, median age was 58 years (range, 40–72 years). Clinical presentation varied according to the location of pseudoaneurysm. Most common manifestations included chest pain ( *n* = 1), sepsis ( *n* = 3), and heart failure ( *n* = 2). Descending aortic pseudoaneurysms were asymptomatic and were identified as incidental findings on computed tomography. The location of the pseudoaneurysm was involving the proximal suture line in one patient and the distal suture line in four patients with aortic root or ascending aortic pseudoaneurysms. Infection was present in four patients and two patients had a history of endocarditis. Positive blood cultures were present in two patients.

### Operative Characteristics

Table 1 demonstrates the operative findings of patients as well as the interval period between the initial operation and development of the pseudoaneurysm. Patients 1 to 5 were all approached through the traditional open surgical method, while patients 6 to 9 underwent device deployment under fluoroscopic guidance. Most of the patients (7 of 9) had a pseudoaneurysm of the ascending aorta and only two patients had pseudoaneurysms of the descending aorta after previous coarctation repair.

### Postoperative Characteristics

Two patients died during the period of the study. Patient 1 died from massive hemorrhage at the site of prior stenting. Patient 7 died from postoperative cardiac arrest and respiratory failure. A single patient required hemorrhage-related reexploration. No patients experienced stroke or acute renal failure following repair. Median hospital and intensive care unit length of stay were 7.1 (range, 1–20) and 2.0 (range, 1–5), respectively.

## Discussion

### Indications for Repair

Most patients present with symptoms of chest pain, heart failure or symptoms related to sepsis. In our series, the main indications to intervene were symptoms and risk of impending rupture. 1 Size of the pseudoaneurysm was not used as a criterion for surgical intervention in our study. Although an incidental finding of pseudoaneurysm of descending aorta as a result of previous coarctation repair may occur, most patients were symptomatic. Common causes of pseudoaneurysms include leak at the anastomotic or cannulation sites, especially in patients with previous dissection repairs. Most patients are young but have had previous cardiovascular surgery; many have endocarditis. Most of our patients required ascending aorta and/or aortic root replacement.

### Technical Challenges of Repair

Repair of pseudoaneurysms of the aorta is often complicated and associated with significant morbidity and mortality, especially when the pseudoaneurysm is in close proximity to the underside of the sternum. Open repair of these lesions may still be approached through a median sternotomy; however, some hypothesize that those more closely related to the sternum may be better candidates for a right anterior thoracotomy or a minimally invasive “J” incision. 1 However, measures were often taken to ensure safety. Cardiopulmonary bypass, for example, may be initiated before sternal entry in patients with a pseudoaneurysm lying in close approximation to the sternum. The pseudoaneurysm for patient 3 in our cohort was punctured upon initial entry into the chest requiring immediate initiation of cardiopulmonary bypass. The remaining three patients that had pseudoaneurysms abutting the sternum all underwent cardiopulmonary bypass via the femoral vessels prior to sternal reentry.

### Percutaneous Repair: Pitfalls and Limitations

**Fig. 1:**
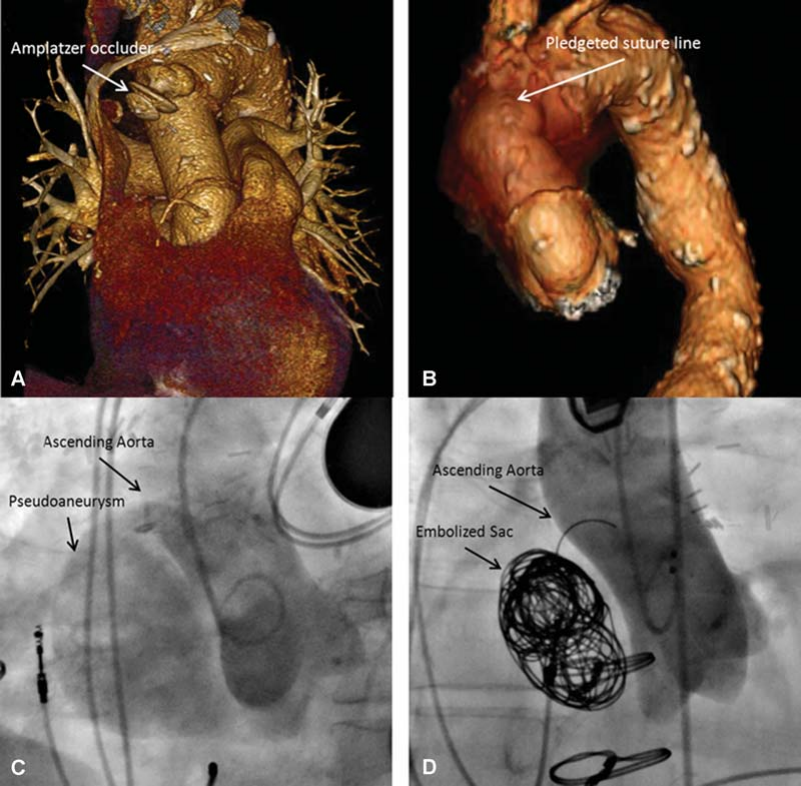
( **A** ) Extruded occluder device into the peudoaneurysm sac requiring reintervention. ( **B** ) The occluder and aneurysm sac were removed surgically and the incision was reapproximated with felt strips. ( **C** ): Pseudoaneurysm successfully excluded after Amplatz's excluder deployment. ( **D** ) Repair of ascending aortic pseudoaneurysm with coil embolization prior to percutaneous occlusion device placement.

In the high risk setting, a minimally invasive approach using endovascular grafts or atrial septal occlusion devices can ameliorate some risks associated with open procedures, while providing adequate exclusion of the pseudoaneurysm sac. However, percutaneous devices are not without limitation. One requirement for occlusion device placement is a narrow neck between the pseudoaneurysm and the aorta to maintain adequate device seal upon deployment. Also, coil embolization can be useful to help achieving stasis within the pseudoaneurysm sac prior to device deployment; however, too many coils can push the device out of the sac, as was the case for patient 5. The situation necessitated an open approach with pseudoaneurysm resection less than 1 year following the prior repair ( Fig. 1 ). In contrast, patient 6 underwent coil embolization of the pseudoaneurysm sac ( Fig. 1 ) prior to occluder placement and has since been asymptomatic.

Endovascular grafts have also been used for high-risk patients with device deployment over the orifice of the lesion. 5 Patients 7 to 9 underwent successful endovascular repair of pseudoaneurysms that would have either required a significantly more complicated open surgical route than the traditional approach or were better candidates for the endovascular approach due to the location of the lesion. For example, patients 8 and 9 both exhibited lesions in the descending aorta where adequate covering of the pseudoaneurysm was fairly easier to achieve without impinging upon other vessels. The pseudoaneurysm in patient 7 resided in close approximation to the manubrium. In this case, endograft deployment allowed for exclusion of the pseudoaneurysm without a median sternotomy. Often however, complete coverage of the lesion along with adequate proximal and distal sealing zones may be difficult to achieve when the pseudoaneurysm arises near the origin of the head vessels. Endovascular approach is often more feasible in patients with pseudoaneurysms of the descending aorta, as compared with those of the ascending aorta given the presence of adequate proximal and distal landing zone.

## Conclusions

Our review demonstrates the challenges of repairing pseudoaneurysms and the variety of treatment modalities effective in repairing these lesions. We conclude that pseudoaneurysm repair can be effectively achieved through open or percutaneous repair but only after careful consideration of anatomical constraints, as well as patient comorbidities.

## References

[1] Atik F A Navia J L Svensson L G Surgical treatment of pseudoaneurysm of the thoracic aorta J Thorac Cardiovasc Surg 2006 132 02 379 385 16872966 10.1016/j.jtcvs.2006.03.052

[2] Katzenschlager R Ugurluoglu A Ahmadi A Incidence of pseudoaneurysm after diagnostic and therapeutic angiography Radiology 1995 195 02 463 466 7724767 10.1148/radiology.195.2.7724767

[3] Kpodonu J Wheatley G H III Ramaiah V G Rodriguez-Lopez J A Strumpf R K Diethrich E B Endovascular repair of an ascending aortic pseudoaneurysm with a septal occluder device: mid-term follow-up Ann Thorac Surg 2008 85 01 349 351 18154851 10.1016/j.athoracsur.2007.06.053

[4] Chapot R Aymard A Saint-Maurice J P Bel A Merland J J Houdart E Coil embolization of an aortic arch false aneurysm J Endovasc Ther 2002 9 06 922 925 12546598 10.1177/152660280200900630

[5] Schwill S LeMaire S A Green S Y Bakaeen F G Coselli J S Endovascular repair of thoracic aortic pseudoaneurysms and patch aneurysms J Vasc Surg 2010 52 04 1034 1037 20619593 10.1016/j.jvs.2010.04.078

